# Biodegradation of the metallic carcinogen hexavalent chromium Cr(VI) by an indigenously isolated bacterial strain

**DOI:** 10.4103/1477-3163.63584

**Published:** 2010-05-20

**Authors:** Alok Prasad Das, Susmita Mishra

**Affiliations:** Centre of Biotechnology, Siksha ‘O’ Anusandhan University, Bhubaneswar, India; 1 Department of Chemical Engineering, National Institute of Technology, Rourkela, Orissa, India

**Keywords:** Biodegradation, *Brevebacterium casei*, carcinogen, hexavalent chromium, mutagenic, 16S rRNA sequencing

## Abstract

**Background:**

Hexavalent chromium [Cr(VI)], a potential mutagen and carcinogen, is regularly introduced into the environment through diverse anthropogenic activities, including electroplating, leather tanning, and pigment manufacturing. Human exposure to this toxic metal ion not only causes potential human health hazards but also affects other life forms. The World Health Organization, the International Agency for Research on Cancer, and the Environmental Protection Agency have determined that Cr(VI) compounds are known human carcinogens. The Sukinda valley in Jajpur District, Orissa, is known for its deposit of chromite ore, producing nearly 98% of the chromite ore in India and one of the prime open cast chromite ore mines in the world (CES, Orissa Newsletter).

**Materials and Methods:**

Our investigation involved microbial remediation of Cr(VI) without producing any byproduct. Bacterial cultures tolerating high concentrations of Cr were isolated from the soil sample collected from the chromite-contaminated sites of Sukinda, and their bioaccumulation properties were investigated. Strains capable of growing at 250 mg/L Cr(VI) were considered as Cr resistant.

**Results:**

The experimental investigation showed the maximum specific Cr uptake at pH 7 and temperature 30°C. At about 50 mg/L initial Cr(VI) concentrations, uptake of the selected potential strain exceeded 98% within 12 h of incubation. The bacterial isolate was identified by 16S rRNA sequencing as *Brevebacterium casei.*

**Conclusion:**

Results indicated promising approach for microbial remediation of effluents containing elevated levels of Cr(VI).

## INTRODUCTION

Environmental pollution due to chromium (Cr) and its compounds is because of a large number of industrial operations, including mining, chrome plating, pigments, petroleum refining, leather tanning, wood preserving, textile manufacturing, pulp processing, and electroplating industries.[1] It exists both in hexavalent and trivalent forms.[2] However, Cr(VI) is very toxic, carcinogenic, and mutagenic both in humans and animals, whereas Cr(III) is an essential micronutrient for many higher organisms.[3] Sukinda valley of Orissa contains 98% of India’s chromite ore deposits and one of the prime open cast chromite ore mines of the world (Centre for Environmental Studies, *Orissa Newsletter*). Mining activity in this region generates around 7.6 million tonnes of solid waste in the form of rejected minerals, overburden material/waste rock, and subgrade ore. Due to the seepage of water from the dumped wastes, the nearby water stream gets contaminated with Cr(VI) at a concentration much above the permissible limits. The Orissa Voluntary Health Association reported health hazards due to Cr(VI) contamination leading to death in few cases. The main diseases include gastrointestinal bleeding, tuberculosis, asthma, infertility, birth defects, and stillbirths. Currently, the effluents are treated with ferrous sulfate, chemical reduction, followed by alkaline precipitation or removal by ion exchange; however, the adsorption that suffers from precipitation and additional treatment methods to remove those are to be sorted. The search for new and innovative technology has drawn the attention on biotransformation of metals by microbes. The engineered use of this detoxification mechanism could be an attractive alternative for the remediation of Cr(VI) pollution. Many microbes have been reported to reduce Cr(VI) under either aerobic or anaerobic conditions with their exceptional ability to adapt to and colonize the noxious metal polluted environments, which are uninhabitable by higher organisms. These microorganisms have developed the capabilities to protect themselves from heavy metal toxicity by various mechanisms, such as adsorption, uptake, methylation, oxidation, and reduction. However, the availability of effective Cr(VI)-reducing organisms is an essential prerequisite for the bioreduction-based remediation of Cr(VI)-contaminated water/soil.

Recently, bioremediation of Cr(VI) has gained considerable consideration.[4,5] Some microbial species can use Cr(VI) as a terminal electron acceptor in their respiratory process and transform Cr(VI) to less toxic Cr(III) compounds.[6,7] A number of these microorganisms, particularly bacteria, can reduce Cr and therefore detoxify it.[8] The present study describes a microbiological treatment for industrial effluent that may be suitable for processing Cr-contaminated waste. This study proposes a remediation route for detoxification of Cr(VI) using an indigenous microorganism.

## MATERIALS AND METHODS

### Isolation and culture conditions

For the isolation of Cr-resistant bacteria, 1 g of the soil sample was serially diluted and plated on PYE medium (Peptone, Yeast extract) agar plates containing 100 *μ*g of Cr^6+^/mL supplemented as K_2_Cr_2_O_7_ to the medium. The growth of the bacterial colonies was observed after 24 h of incubation at 30°C. Isolated colonies were picked up with sterilized wire loop and streaked on PYE agar medium plate containing 100 mg/mL Cr^6+^.

### Sample characterization

*The* soil samples collected (0–15 cm depth) from different locations of the Cr deposited and contaminated site located at Sukinda mines, Jajpur, Orissa, were examined to estimate the amount of pollutants in the samples. Isolation of cultures from the soil sample collected was carried out as per standard procedures.[9] The physical and chemical properties, such as soil organic matter, Cr(VI), total Cr, pH of the soil samples obtained from these waste sites were characterized. The presence of other heavy metals was estimated by Atomic Absorption Spectroscopy methods.

### Scanning electron microscopy–energy-dispersive X-ray microanalysis for elemental analysis of the soil sample

Energy-dispersive X-ray microanalysis (EDX) is a powerful technique that allows the qualitative and quantitative measurement of many elements of physiologic interest at the cellular and subcellular level. Scanning electron microscopy–EDX (SEM–EDX) was used to understand the morphology, elemental composition, and particle density of the Cr-contaminated soil sample to further investigate the potential sources as well as transport of the pollutants. The collected soil sample was mounted on electron microprobe stubs. The SEM–EDX analyses were carried out with the help of a computer-controlled field emission SEM (JEOL JSM-6330F, JEOL Ltd., Akishima, Tokyo, Japan) equipped with an EDX detection system.

### Nutrient media

Bacterial strains, resistant to Cr(VI), were isolated from the soil using the serial dilution technique in PYE medium. Agar supplemented with 100 g Cr(VI)/mL as K_2_Cr_2_O_7_ and 0.5% (wt/vol) glucose as a carbon source. The pH was maintained at 7 ± 0.2 by using HCl or NaOH. The isolates were tested for their chromate tolerance at different concentrations (12.5, 25, 50, 75, 100 *¼*L/mL) of Cr(VI) supplemented as K_2_Cr_2_O_7_. Significant growth of the specific bacterial species in the presence of 100 mg Cr(VI)/L in PYE medium during 2-day incubation at 30°C, were considered as Cr(VI) resistant. A single strain was capable of growing at this condition and was selected for further experiments.

### Culture identification

Five milliliters of pure culture overnight and spin down at 3000 rpm. Genomic DNA was isolated from the pure culture pellet. Using consensus primers, the ~1.5-kb 16S rDNA fragment was amplified using high-fidelity PCR polymerase. Sequence data were aligned and analyzed for finding the closest homology for the microbe based on the nucleotides’ homology and phylogenetic analysis 16S rRNA sequencing.

**Figure 1:**
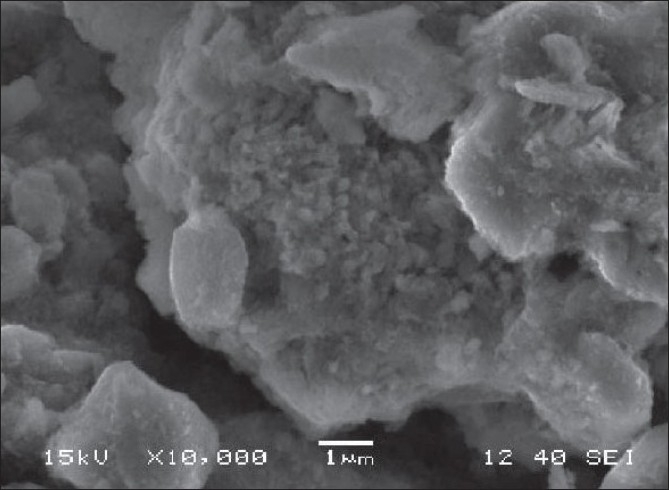
Scanning electron micrograph of soil sample

### Process optimization

The optimization study was carried out for a duration of 48 h. Both for temperature and pH optimization, the experiments were carried out in triplicates. For temperature optimization, the 5 standard temperatures considered were 26, 28, 30, 32, and 34°C, whereas for pH optimization, the 5 standards used were 4, 5, 6, 7, 8, and 9. The respective pH's were adjusted with conc. NaOH and conc. HCl.

### Cr(VI) reduction

The bacterial strain was precultured overnight in PYE broth. Culture flasks (150 mL with a final liquid volume of 30 mL) containing minimal salt agar medium supplemented with Cr(VI) ranging from 50–100 mg Cr(VI)/L medium, and 0.5% glucose were inoculated with equal amounts of culture species. Media without Cr(VI) were inoculated with bacteria, and uninoculated media containing Cr(VI) served as controls. All the cultures, including controls (in duplicate) were incubated for 24 h at room temperature (30°C) with a shaking speed of 100 rpm. Growth of the bacteria was monitored at specific time intervals by measuring the optical density of the cultures at 600 nm. To compute the Cr(VI) reduction by growing cells, a 1-mL culture from each of the above-mentioned flasks was centrifuged (6000 rpm for 10 min at 10°C) and the supernatant was analyzed for Cr(VI).

**Figure 2:**
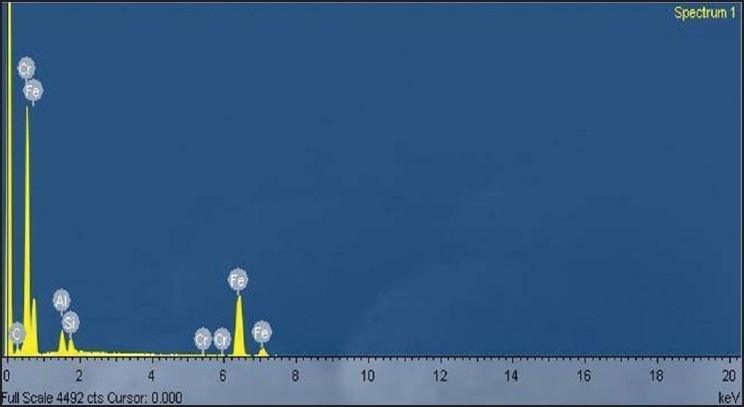
Energy-dispersive X-ray microanalysis of the soil sample

## RESULTS AND DISCUSSION

### Screening of microorganisms for chromium resistance

**Figure 3:**
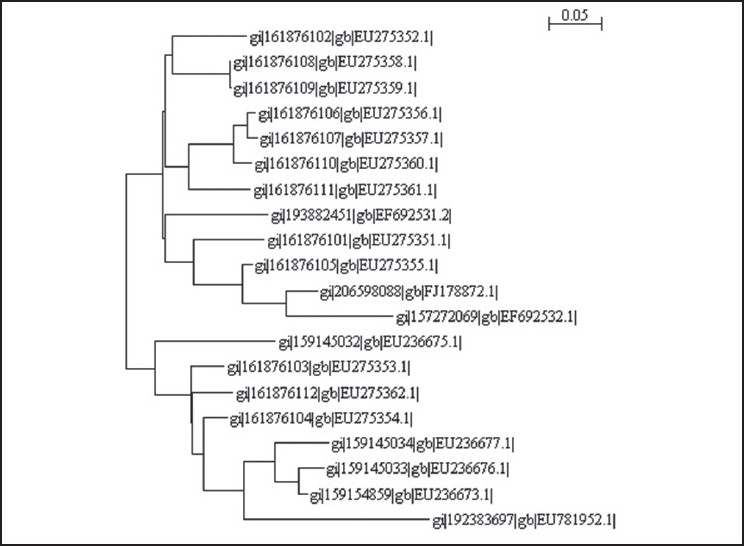
Phylogenetic tree made in MEGA 3.1 software using neighbor joining method—optimum parameters for Cr(VI) bioreduction

The isolates were tested for their chromate tolerance at different concentrations (20–200*μ*L/mL) in solid agar medium. Three bacteria showed resistance to 100 mg/L of Cr(VI) in nutrient agar media among which *Brevibacterium* sp. was able to grow to a concentration of 250 mg/L of Cr(VI) as illustrated in Figure 3. Two isolated species, such as *Arthrobacter* sp. and a *Bacillus* sp., from tannery waste contaminated soil that showed similar resistance to Cr(VI) and had the ability to reduce Cr(VI) to Cr(III).[10] Both the bacterial strains tolerated for Cr(VI) at 100 mg/mL on a minimal salt agar medium supplemented with 0.5% glucose, but only *Arthrobacter* could grow in liquid medium at this concentration. *Arthrobacter* sp. could reduce Cr(VI) up to 50 *μ*g/mL, whereas *Bacillus* sp. was not able to reduce Cr(VI) beyond 20 *μ*g/mL. *Arthrobacter* sp. was distinctly superior to the *Bacillus* sp. in terms of their Cr(VI)-reducing ability and resistance to Cr(VI).

### Physicochemical analysis of the collected samples

Physicochemical parameters of contaminated soil estimated are shown in Table 1. The pH of the samples was determined with an ion-specific electrode and the pH of the soil and waste water samples were in the range of 7.4–7.8. This indicated that the chromite-contaminated sites are slightly alkaline in nature. Cr(VI) in the contaminated soil sample was in the range of 2–6 mg/L. Soil organic matter varied from 5.2 ± 0.5%, total dissolved solids and total suspended solids in the effluent varied form 480–560 mg/L. Our results showed similarity with the results reported elsewhere.[11]

**Table 1:** Physicochemical characteristics of the soil sample

Parameters	Value
Soil organic matter	5.2 ± 0.5%
Total suspended solids	560 mg/L
Total dissolved solids	480 mg/L
Total Cr	9 ± 12 mg/g of soil
Cr (VI)	2 – 5.9 mg/g of soil
pH	7.4 – 7.8

### Scanning electron micrographs and energy-dispersive X-ray microanalysis spectrum

The SEM analysis of soil samples determines its morphologic characteristics. The micrograph illustrates the shape of the soil particles [Figure 1]. Chemical composition was determined by using an EDX spectrometer. Analyses of these soil particles, with EDX, indicate that Cr is present in the soil sample along with other heavy metals, such as Fe, Al, and Si, and is shown in Figure 2. Similar reports of morphologic characteristics and elemental chemical analyses of the soil performed with SEM-EDX collected from the facilities of the Química Central Chromate Factory, which is located at Leon-San Francisco del Rincon.[12]

**Figure 4:**
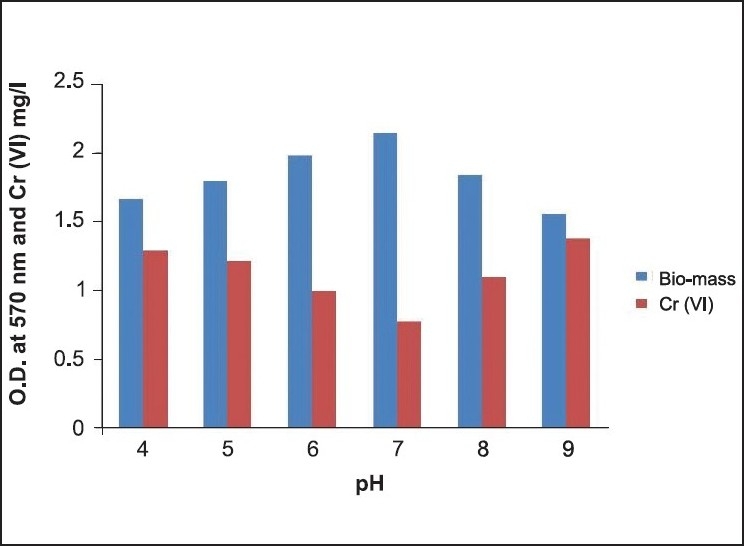
Temperature optimization for Cr(VI) degradation

**Figure 5:**
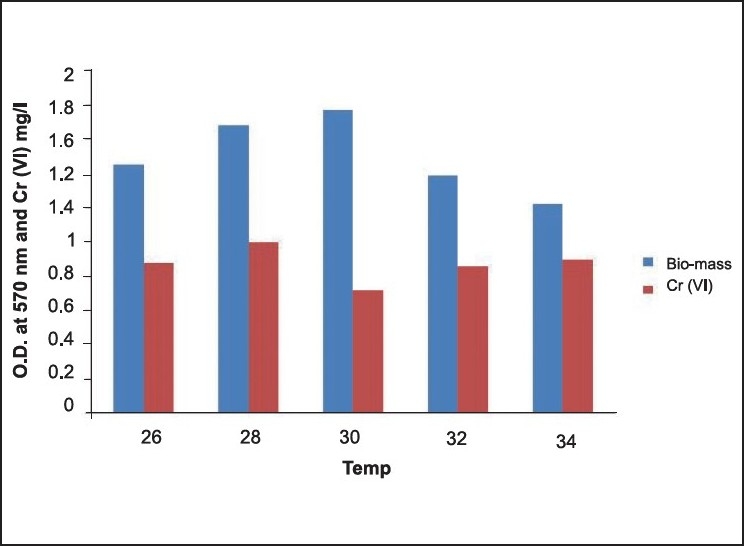
pH optimization for Cr(VI) degradation

**Figure 6:**
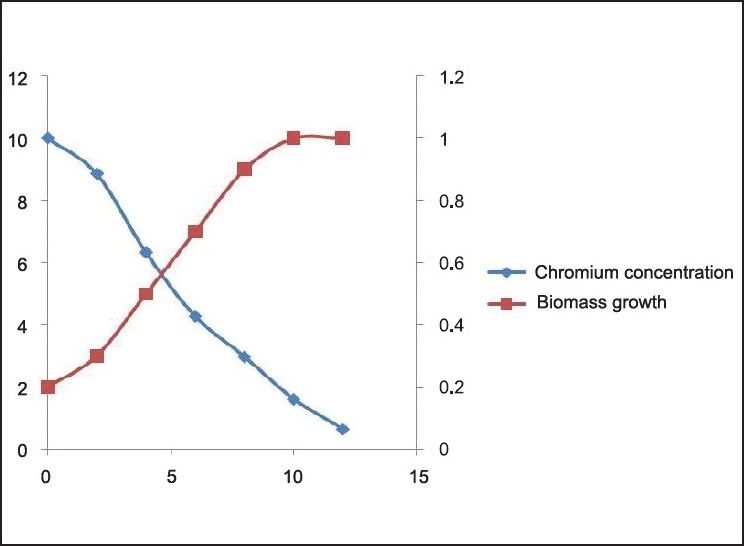
Cr (VI) degradation kinetics and Biomass growth varying initial chromium concentration 10mg/L

### 16S rRNA identification and phylogenetic analysis of the enrichment culture

Based on the nucleotide homology and phylogenetic analysis, the microbe was detected to be *Brevibacterium* casei [GenBank Accession Number: EU781952; Figure 3].

Cr(VI) degradation is significantly affected by various process parameters, such as initial pH and incubation temperature. At optimum pH 7, Cr(VI) degraded from 50-0.76 mg/L reducing Cr(VI) by 98%. Optimal pH for growth of Cr(VI)-resistant bacteria was evidenced at 7.0–7.8.[13,14] The effect of pH on Cr(VI) reduction by the bacterial strain *Brevebacterium* casei cultures were initially supplied with 50 mg/L of Cr(VI) with inoculum volume of 1 mL. The chromate reduction study was carried out using freshly prepared overnight culture incubated at 30°C with shaking at 200 rpm. The cultures were harvested after a 12–h incubation period. Using B. casei strain, Cr(VI) reduction occurred at a pH range of 4–9, but an optimum reduction was observed at pH 7. pH was adjusted with 1 M NaOH and 1 M HCl. It was observed from the experiment that the extreme pH (4 and 9) restricted bacterial growth and Cr(VI) reduction. pH and Cr(VI) reduction relationship was not surprising because chromate (CrO_4_^2−^) is the dominant Cr(VI) species in an aqueous environment at pH 6.5–9.0.[16] However, because Cr(VI) reduction is enzyme-mediated, variation in pH will affect the degree of ionization of the enzyme, changing the protein′s conformation and affecting the enzyme activity. [18] At optimum pH 7, Cr(VI) degraded from 50–0.78 mg/L reducing Cr(VI) by 98%. Optimal pH for growth of Cr(VI)-resistant bacteria was evidenced at 7.0-7.8[13,17]; Laxman *et al*. (2007) also reported that an optimum pH of 6-7 is needed for the reduction of hexavalent Cr by *Streptomyces griseus*. Again Donati *et al*. (2003) suggested that the cultures at pH 6.0 and 7.0 showed lag phases shorter than those at pH 5.0. At pH 6.0, cultures had the highest free bacterial populations and the highest Cr reduction values. It is demonstrated that maximum Cr(VI) degradation occurred at 30°C reducing Cr(VI) from 50–0.6 mg/L[13,15] at an optimal temperature of 30–37°C for Cr(VI) reduction. As evident from the experimental values, the suitable temperature and pH for hexavalent Cr reduction are 30°C and 7, respectively [Figures 4, 5].

**Figure 7:**
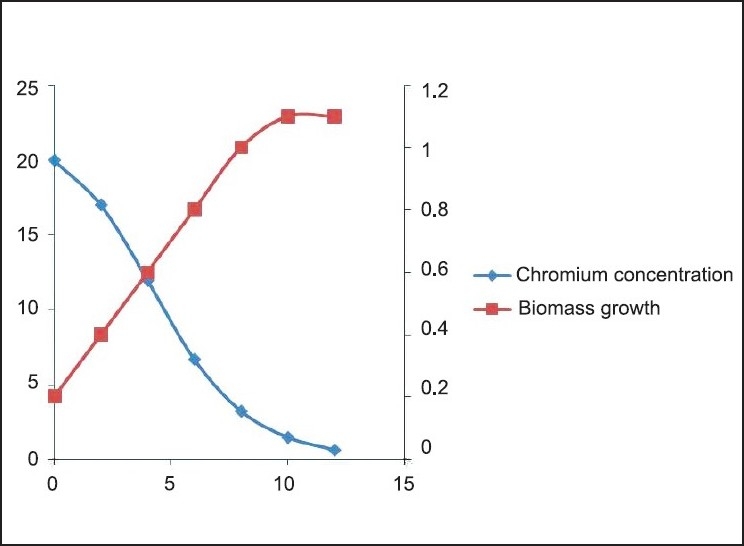
Cr (VI) degradation kinetics and Biomass growth varying initial chromium concentration 20 mg/L

**Figure 8:**
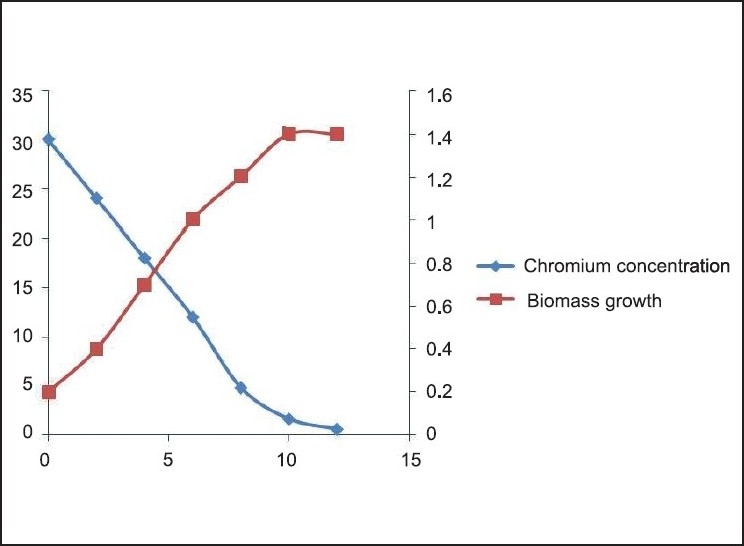
Cr (VI) degradation kinetics and Biomass growth varying initial chromium concentration 30 mg/L

**Figure 9:**
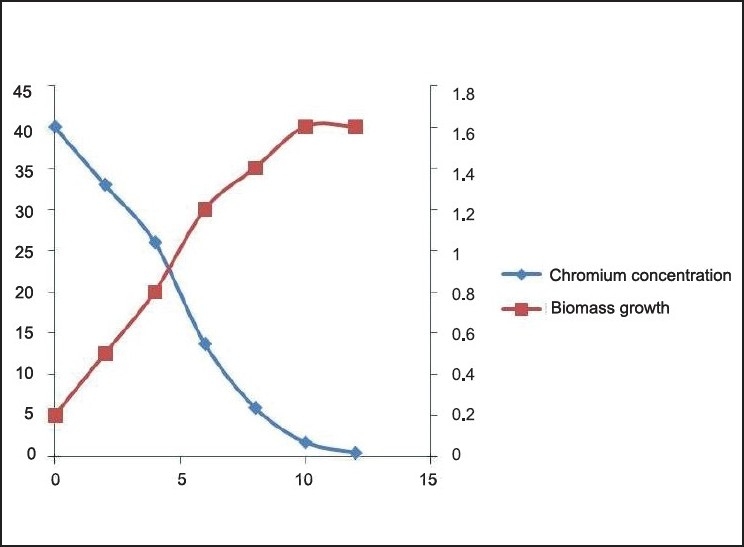
Cr (VI) degradation kinetics and Biomass growth varying initial chromium concentration 40 mg/L

**Figure 10:**
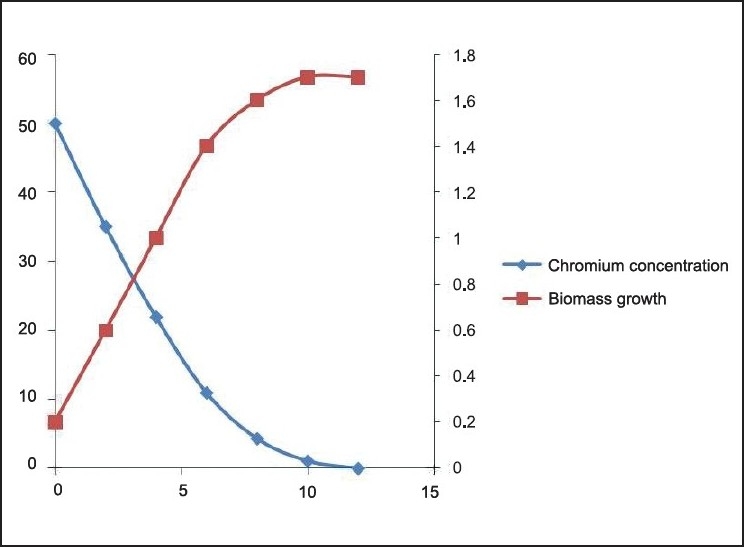
Cr (VI) degradation and Biomass growth with initial concentration 50mg/l

**Figure 11:**
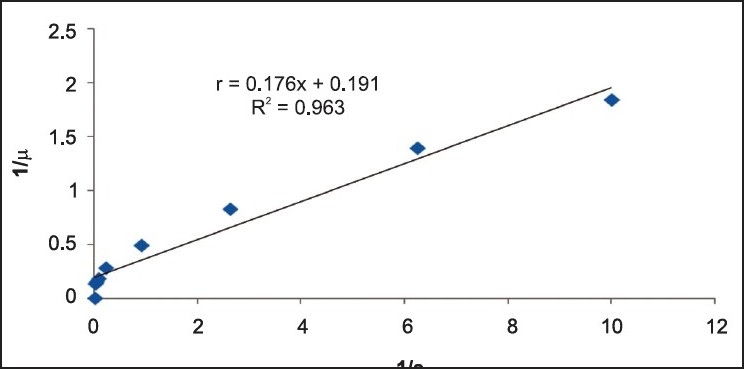
Michael-Menten plot for B.Casei at Cr (VI) concentration 50 mg/L

### Measurement of the kinetics of broth cellular growth Cr(VI) reduction

The Cr(VI) reduction ability of the bacteria was growth dependent and the *Brevibacterium* sp. reduces nearly 50 mg/L of Cr(VI) during the degradation experiment in 12 h [Figure 10]. It is evident from the experiment that the time required for Cr(VI) degradation varies with initial Cr concentration. We can also find 3 different stages of degradation with initial rapid stage followed by a slow rate and finally degradation at a much slower rate. Initial rapid degradation is observed within 8 h for varying Cr concentrations. At lower Cr concentrations, equilibrium is achieved within 12 h with 99% removal. Hence, we can see that maximum reduction is observed within 12 h at an initial Cr concentration of 50 mg/L. This is attributed to the high concentration gradient of Cr in the solution, which is the main driving force for Cr(VI) degradation. The inoculum of the bacterial strains cultured overnight was used for this experiment. Culture flasks (150 mL) with a final volume of 100 mL supplemented with (10–40 mg/L) of Cr(VI) were inoculated with 2 mL of inoculums for 24 h. The growth kinetics of bacteria was characterized as initial lag phase, second exponential phase, stationary phase, and death phase. In this experiment, it was observed that the lag phase was increasing with increased initial Cr(VI) concentration [Figures 6–9]. It is basically due to the inhibitory effect of higher Cr concentrations on the growth of the organism. Each organism has a specific resistance at a specified growth condition. As the initial age of the inoculums was fixed at 24 h, the acclimatization period at varying Cr concentrations would not remain the same. Hence, the following behavior was observed. The Cr-resistant bacterial isolate showed reduced bioaccumulation when the cells were in stationary phase. At higher concentrations, the growth of the bacteria was inhibited due to a fixed amount of inoculums for all the different concentration levels of Cr(VI) considered in the experiment.

Michaelis-Menten kinetics is used to describe the kinetics of various biological species. This kinetic model is relevant to situations where very simple kinetics can be assumed. The Michaelis-Menten equation relates the initial reaction rate *μ* to the substrate concentration (S). The equation can be represented as follows:

μ=μmax(S)S+Km

Figure 11 shows the Michaelis–Menten plot between *μ* versus S at an initial Cr(VI) concentration of 50 mg/L. The Michaelis *constant (Km)* and maximum growth rate (*μ*_max_) values are estimated as 1.018 and 6.06 g/L/h, respectively.

## CONCLUSION

In this study, a newly isolated Cr(VI)-reducing bacterium was identified as a *Brevibacterium* sp. This bacterium reduced Cr(VI) anaerobically at the expense of PYE as the source for growth. The experimental results concluded the existence of Cr-resistant bacterial strains isolated from anthropogenically Cr-percolated ecosystems. It is seen that the identified species *Brevibacterium casei* can effectively degrade Cr(VI) up to 99% in 12 h at neutral pH and a temperature of 30°C. These findings are potentially useful because this bacterium could be harnessed to the detoxification of chromate-contaminated industrial and mining waste. This needs further research as the resistance potential of these organisms indicates the possibility of their exploitation in Cr and other heavy metal bioremediation in the future.

## AUTHOR'S PROFILE

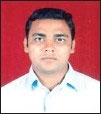



Mr. Alok Prasad Das Lecturer Center of Biotechnology SOA University, Bhubaneswar E-mail: alok1503@gmail.com Mob.No:+919337048198. 1. Coinvestigator of an R & D project funded (Rs 75,000/-) by Institute of Engineers India entitled, “Isolation & Characterization of chromium-resistant and -reducing bacteria in a chromium-contaminated site”. Ref. No.: SCK/T-R&D/56/2006-2007. Research Areas: ŀEnvironmental Bioremediation of contaminated soils and water with heavy metals. ŀDomestic, Industrial & Mining wastewater treatment with emphasis on wastewater reuse and recycling. ŀMicrobial Genomics. ŀFermentation Technology, Bioprocess Technology
